# Optogenetic stimulation of striatal patches modifies habit formation and inhibits dopamine release

**DOI:** 10.1038/s41598-021-99350-5

**Published:** 2021-10-06

**Authors:** J. A. Nadel, S. S. Pawelko, J. R. Scott, R. McLaughlin, M. Fox, M. Ghanem, R. van der Merwe, N. G. Hollon, E. S. Ramsson, C. D. Howard

**Affiliations:** 1 Neuroscience Department, Oberlin College, Oberlin, OH USA; 2 Molecular Neurobiology Laboratory, The Salk Institute for Biological Studies, La Jolla, CA USA; 3 Department of Biomedical Science, Grand Valley State University, Allendale, MI USA

**Keywords:** Motivation, Neural circuits, Reward, Neuroscience, Operant learning

## Abstract

Habits are inflexible behaviors that develop after extensive repetition, and overreliance on habits is a hallmark of many pathological states. The striatum is involved in the transition from flexible to inflexible responding, and interspersed throughout the striatum are patches, or striosomes, which make up ~15% of the volume of the striatum relative to the surrounding matrix compartment. Previous studies have suggested that patches are necessary for normal habit formation, but it remains unknown exactly how patches contribute to habit formation and expression. Here, using optogenetics, we stimulated striatal patches in Sepw1-NP67 mice during variable interval training (VI60), which is used to establish habitual responding. We found that activation of patches at reward retrieval resulted in elevated responding during VI60 training by modifying the pattern of head entry and pressing. Further, this optogenetic manipulation reduced subsequent responding following reinforcer devaluation, suggesting modified habit formation. However, patch stimulation did not generally increase extinction rates during a subsequent extinction probe, but did result in a small ‘extinction burst’, further suggesting goal-directed behavior. On the other hand, this manipulation had no effect in omission trials, where mice had to withhold responses to obtain rewards. Finally, we utilized fast-scan cyclic voltammetry to investigate how patch activation modifies evoked striatal dopamine release and found that optogenetic activation of patch projections to the substantia nigra pars compacta (SNc) is sufficient to suppress dopamine release in the dorsal striatum. Overall, this work provides novel insight into the role of the patch compartment in habit formation, and provides a potential mechanism for how patches modify habitual behavior by exerting control over dopamine signaling.

## Introduction

Organisms must optimize action patterns to be successful in their environments. This optimization process can come in two forms: updating of actions can be highly flexible and dependent on outcomes (action-outcome, or goal-oriented strategies), or action updating can be resistant to change regardless of outcome (stimulus–response or habitual strategies)^1^. Habitual, automated behaviors can be highly advantageous, as they allow animals to respond to stimuli without great cognitive effort. However, habits can also present as maladaptive behaviors that persist in spite of negative outcomes. Dysfunctional habit formation underlies many pathological states, including drug addiction^2^.

In animal models, habits have been studied by measuring perseverance of instrumental behaviors following reduction in reward value, or by measuring flexibility when action-outcome contingencies are manipulated^3,4^. Using these approaches, distinct neural circuits underlying goal-directed and habitual responding have been identified. A well supported model has emerged positing that the dorsomedial striatum is crucial for goal-directed behaviors, while the dorsolateral striatum is necessary for the development of habitual behaviors^5–7^. Similarly, specific patterns of corticostriatal plasticity in the lateral striatum correlate with habitual responding^8^, and human imaging studies have linked activity in lateral striatum (putamen) with habitual behaviors^9^. However, this model could be somewhat oversimplified, as recent studies suggest the medial striatum also contributes to inflexible behaviors^10,11^.

Adding a layer of complexity to the medial–lateral striatal divide is the existence of neurochemically distinct subcompartments in the striatum: small, labyrinthine islands called patches or striosomes (comprising 15% of striatal volume), and surrounding ‘matrix’ tissue (85% of striatal volume)^12,13^. In addition to unique cellular markers^14^, patch neurons are characterized by unique electrophysiological profiles^15^ and connectivity, notably providing the predominant anatomical and functional striatal input to midbrain dopaminergic neurons^16,17^. Despite extensive work characterizing the structure and connectivity of striatal patches, their role in behavioral regulation is only partially understood. Previous work has suggested a role for striatal patches in reward processing^18–20^, behavioral invigoration^21^, and cost–benefit decision making^22,23^. Additionally, several studies now support the notion that patches may play a role in the transition from flexible to habitual responding. Striatal patches have been recently shown to be necessary for normal habit formation: specific lesions of patch neurons diminish habitual responding following reward devaluation^24^ or following changes in action-outcome contingencies^25^.

In the current study, we employed optogenetics in Sepw1-NP67 mice, a transgenic line with enriched Cre recombinase expression in striatal patches^26^, to selectively target these structures. Patches or patch projections were stimulated at reward retrieval during a variable interval (VI60) schedule of responding, a task used to induce habitual responding^27^. Mice that received patch stimulation displayed elevated rates of responding during VI60 training driven by changes in structure of presses and head entries. Mice that received optogenetic stimulation also displayed reduced lever pressing and head entry rates to a greater extent than YFP controls following reward devaluation, implying modified habit formation. These effects were not attributable to generalized increases in extinction rate in subsequent extinction trials, and patch stimulation drove a small ‘extinction burst’ early in extinction probes, further suggesting goal-directed behavior. On the other hand, patch stimulation did not alter response rates during omission, when mice had to withhold responding to earn rewards. Contrary to a prior study using non-selective electrical self-stimulation^19^, we did not find optogenetic stimulation of patches to be reinforcing in a place preference task, but stimulation of patches did elevate locomotion in an open field. Finally, to investigate how patch activation modifies circuit function, we employed fast-scan cyclic voltammetry to measure striatal dopamine levels in vivo and determined that optogenetic activation of patches suppresses dopamine release driven by electrical stimulation of excitatory inputs. Together, these results provide evidence that patches enhance ongoing behaviors and play a role in habit formation, potentially by modulating striatal dopamine levels.

## Results

### Optogenetic manipulation of striatal patches or projections in variable interval training

**Figure 1:**
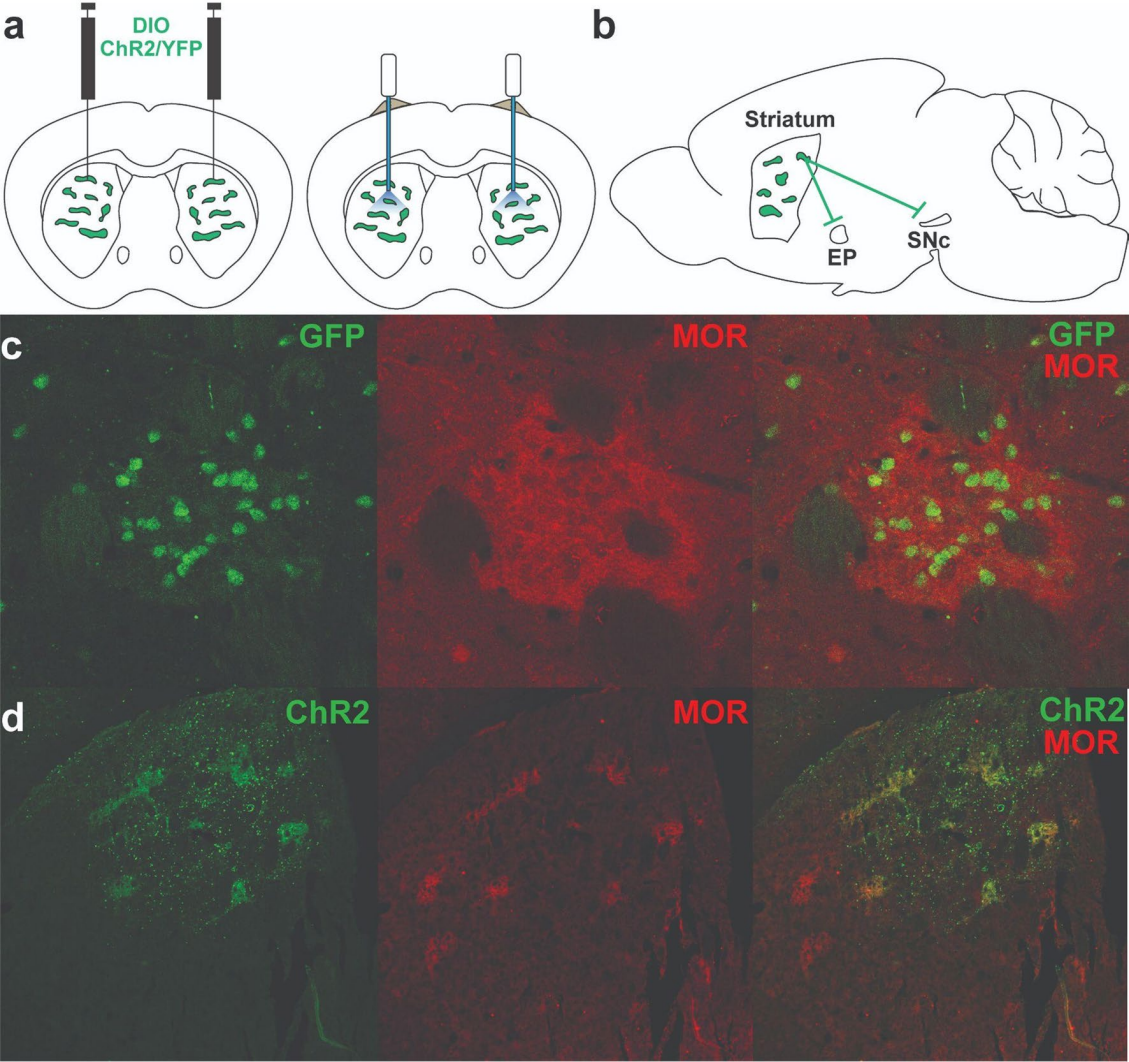
Experimental design and characterization of Sepw1-Cre mice. (**a**) Experimental design. Bilateral AAV driving expression of channelrhodopsin-2 (ChR2) or YFP was injected into the dorsal striatum (left). Fiber optics were affixed just dorsal to injection site (right). (**b**) Patches in striatum, or patch projections to entopeduncular nucleus (EP) or substantia nigra pars compacta (SNc) were targeted with fiber optic implants. (**c**) Coronal section showing Cre dependent expression of GFP and µ-opioid receptor expression demonstrating enriched Cre expression in a striatal patch. (**d**) Coronal section showing AAV5-driven expression of ChR2-eYFP overlaid with µ-opioid receptor expression. (**a** + **b**) drawn in Illustrator CC 2019 (Adobe).

To investigate the role of patch neurons in habit formation, we utilized Sepw1-NP67 mice, which have enriched Cre recombinase expression in striatal patches^26,28^. Crossing these mice with a Cre-dependent GFP reporter line shows enriched GFP + neurons in µ-opiate receptor dense striatal patches (Fig. 1c), though as previously reported, this line also expressed Cre in “exo-patch” neurons, which display similar gene expression and physiological profiles to patch neurons^28^. We injected Sepw1-NP67 mice with an AAV encoding either the Cre-dependent light-gated cation channel ChR2 or YFP in the dorsal striatum, which resulted in enriched ChR2 expression in striatal patches (Fig. 1d). We then implanted fiber optics targeting cell bodies of striatal patch neurons, patch terminals in SNc^16^ or at patch terminals in entopeduncular nucleus^29,30^, (Fig. 1a + b) with the expectation that these two pathways may differentially modulate habitual responding due to potentially opposing effects on dopamine neurons. However, as no implantation site-dependent differences were observed in performance during operant training, responding following devaluation, or in omission, fiber optic placement groups were collapsed into a general “ChR2” group for comparison with YFP controls (individual group data is shown in Supplemental Figures matching main figure numbers; Supplemental Figs. 2, 3). Site-dependent differences were noted in follow-up behavioral tasks (open field and place-preference), suggesting these different output sites may differentially modify aspects of behavior (Fig. 4; see below), so these groups were not collapsed.

**Figure 2:**
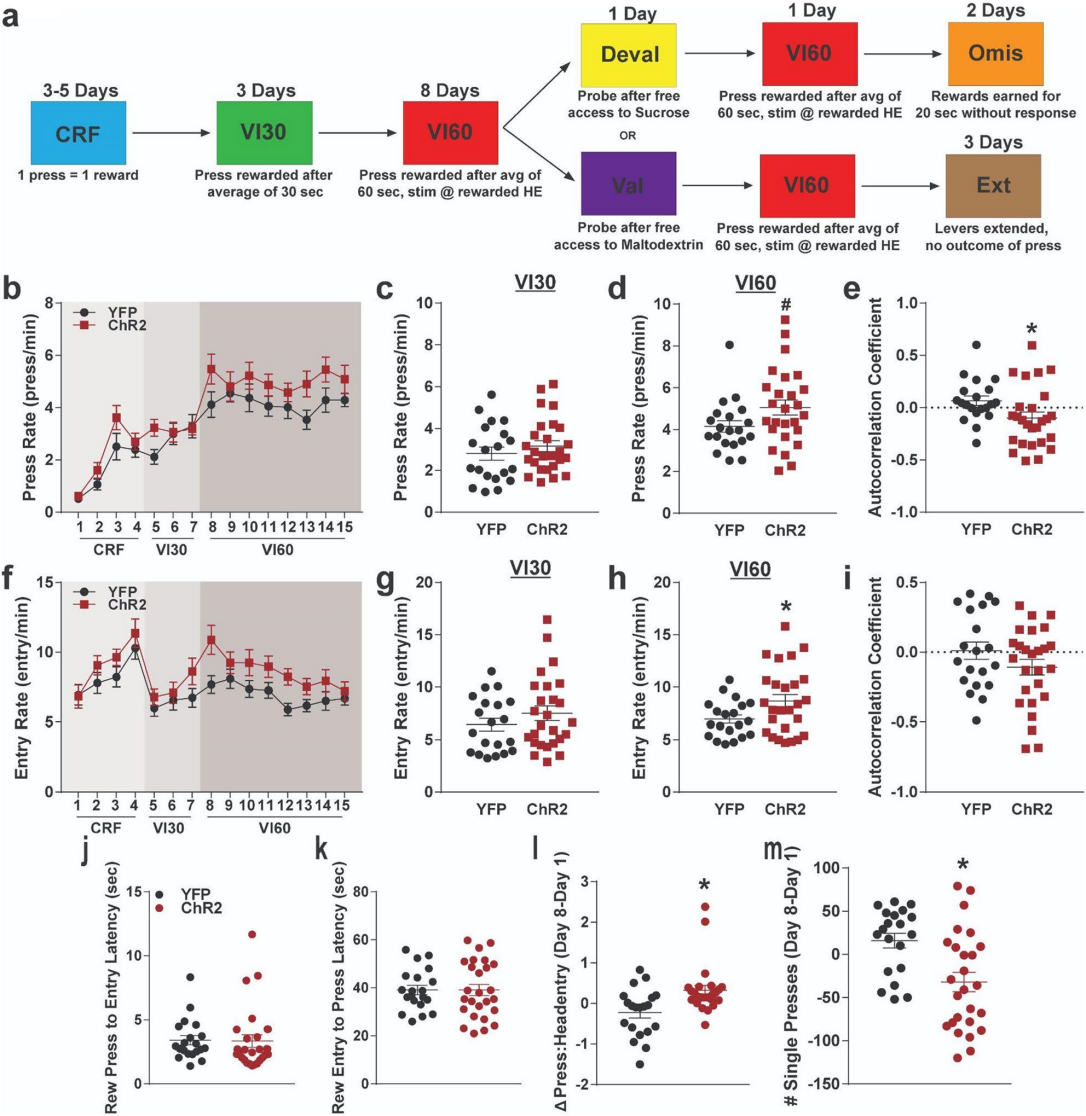
Optogenetic patch stimulation during variable interval training. (**a**) Timeline of behavioral schedule. Two groups of mice were trained on a continuous reinforcement schedule (CRF) before beginning variable interval 30 training (VI30). Following this, mice began a variable interval 60 schedule (VI60), and optogenetic stimulation of patches or patch projections occurred during reward retrieval. One group then underwent devaluation, where mice gained free access to the reinforcer earned during VI60 (10% sucrose) before being tested on a 5 min probe trial. After a day of VI60 retraining, these mice then experienced two days of omission (Omis), where they had to withhold responding to earn rewards. The second group of mice received identical training to the first, but instead of devaluation, these mice received access to an alternative reinforcer (10% maltodextrin) before being tested in a 5 min probe (valuation probe). This group then returned to VI60 training for one day before undergoing two days of extinction, where lever presses yielded no outcome. See Methods for details of each behavioral schedule. (**b**) Press rates for ChR2 and YFP control mice across training. (**c**, **d)** Average response rates across VI30 (**c**) and VI60 (**d**). (**e**) Autocorrelation coefficients for press rates across VI60 training. (**f**) Head entry rates to the food magazine across training for ChR2 and YFP mice. (**g**, **h**) Average head entry rates across VI30 (**g**) and VI60 (**h**). (**i**) Autocorrelation coefficients for entry rates across VI60 training. (**j**–**k**) Latency between rewarded press to next head entry (**j**) and between reward retrieval and next lever press for YFP and ChR2 mice on the final day of VI60 (**k**). (**l**) Change in ratio of presses to head entries from day 1 to day 8 of VI60 training. (**m**) Change in number of single presses between head entries from day 1 to day 8 of VI60 training. *p < 0.05; ^#^*p* < 0.1; error bars, SEM. **a** drawn in Illustrator CC 2019 (Adobe).

Three weeks after surgery, mice were food restricted and trained to depress a lever on a continuous reinforcement schedule (CRF), a variable interval averaging 30 s (VI30), then a variable interval averaging 60 s (VI60) schedule of reinforcement (see Fig. 2a for behavioral schedule), which induces habitual behavior in mice^31^. Beginning in VI60, mice received laser stimulation through fiber optics at reward retrieval (first headentry following reward delivery; 3 s, 5 Hz, 5 mW stimulation). ChR2 and YFP mice both increased press rates across CRF and VI training (two-way repeated measures ANOVA, significant effect of time, F_(6.873, 302.4)_ = 29.24, *p* < 0.0001). Stimulation of patches at reward retrieval also resulted in increased press rates across training for ChR2 mice compared to YFP controls (significant effect of group, F_(1, 44)_ = 4.164, *p* = 0.0473; no significant interaction, *p* > 0.514; Fig. 2b).

This difference was not attributable to differences during VI30 training (t_44_ = 0.9035, *p* = 0.3712; Fig. 2c); rather, this difference emerged during VI60 training, when mice began receiving optogenetic stimulation (t_44_ = 1.883, *p* = 0.0664; Fig. 2d). We previously found that caspase-driven lesions of striatal patches increased response variability across days^25^. Similarly, optogenetic activation of patches during VI60 training significantly reduced press rate consistency as assessed by day-to-day autocorrelation (lag 1 day; unpaired t-test, t_44_ = 2.156, *p* = 0.0366; Fig. 2e). It is possible that changes in press rate could be due to different implantation sites in ChR2 mice; however, we did not note differences in implantation site groups across learning, average VI30, or average VI60 press rates (all *p* ≥ 0.73; Supplemental Fig. 2a–c, see supplemental figure legends for details).

In addition to lever pressing, head entry to the food magazine has been used to assess reward seeking and behavioral flexibility^10,32^. ChR2 mice and YFP controls both altered their entry rates across training (two-way repeated measures ANOVA, significant effect of time, F_(6.600,290.4)_ = 9.122, *p* < 0.0001), though ChR2 mice tended to have higher entry rates (trending effect of group, F_(1, 44)_ = 3.538, *p* = 0.0666; no significant interaction, *p* = 0.440; Fig. 2f). This difference was also not attributable to VI30 (t_44_ = 1.141, *p* = 0.2599; Fig. 2g), but was specific to stimulated VI60 trials (t_44_ = 2.158, *p* = 0.0364; Fig. 2h). Day-to-day variability was not as clear in head entry rates based on autocorrelation (lag 1 day; unpaired t-test, t_44_ = 1.409, *p* = 0.1659; Fig. 2i). Differences in entry rate were also not attributable to implantation site (all *p* ≥ 0.8612; Supplemental Fig. 2d–f). Taken together, these data suggest that stimulation of patches elevates press and entry rates during VI60 when stimulation is paired to rewarded head entries, and that modulation of patches impairs day-to-day press rate consistency.

Patch stimulation may elevate response rates by acutely increasing the speed of transitions between presses and head entries during VI60 trials, or by modifying the sequence of behavioral events during VI60. To distinguish between these possibilities, we compared the time between rewarded presses and reward retrieval, as well as the time between reward retrieval (when optogenetic stimulation occurs) to the next press. Patch stimulation did not alter either of these metrics relative to YFP controls by the final day of VI60 training (unpaired t-test, *p* ≥ 0.926; Fig. 2j and k). We then assessed how patch stimulation changed the structure of responding during VI60 by determining the number of presses that occurred between each head entry and comparing these values between days 1 and 8 within stimulation groups. We found that patch stimulation drove a significant increase in the number of presses per head entry across the eight days of VI60 training (t_44_ = 3.092, *p* = 0.0034; Fig. 2l). This change occurred concurrently with a decrease in the number of single presses between head entries across VI60 (t_44_ = 3.222, *p* = 0.0024; Fig. 2m), suggesting that on average, YFP and ChR2 mice differently modify the number of lever presses during press bouts between head entries. Overall, these data suggest that activating striatal patches across training biases the structure of responding towards increased presses per bout as opposed to invigorating the rate of transitions between behavioral events.

### Characterizing patch stimulation during learning in habit and extinction probes

**Figure 3:**
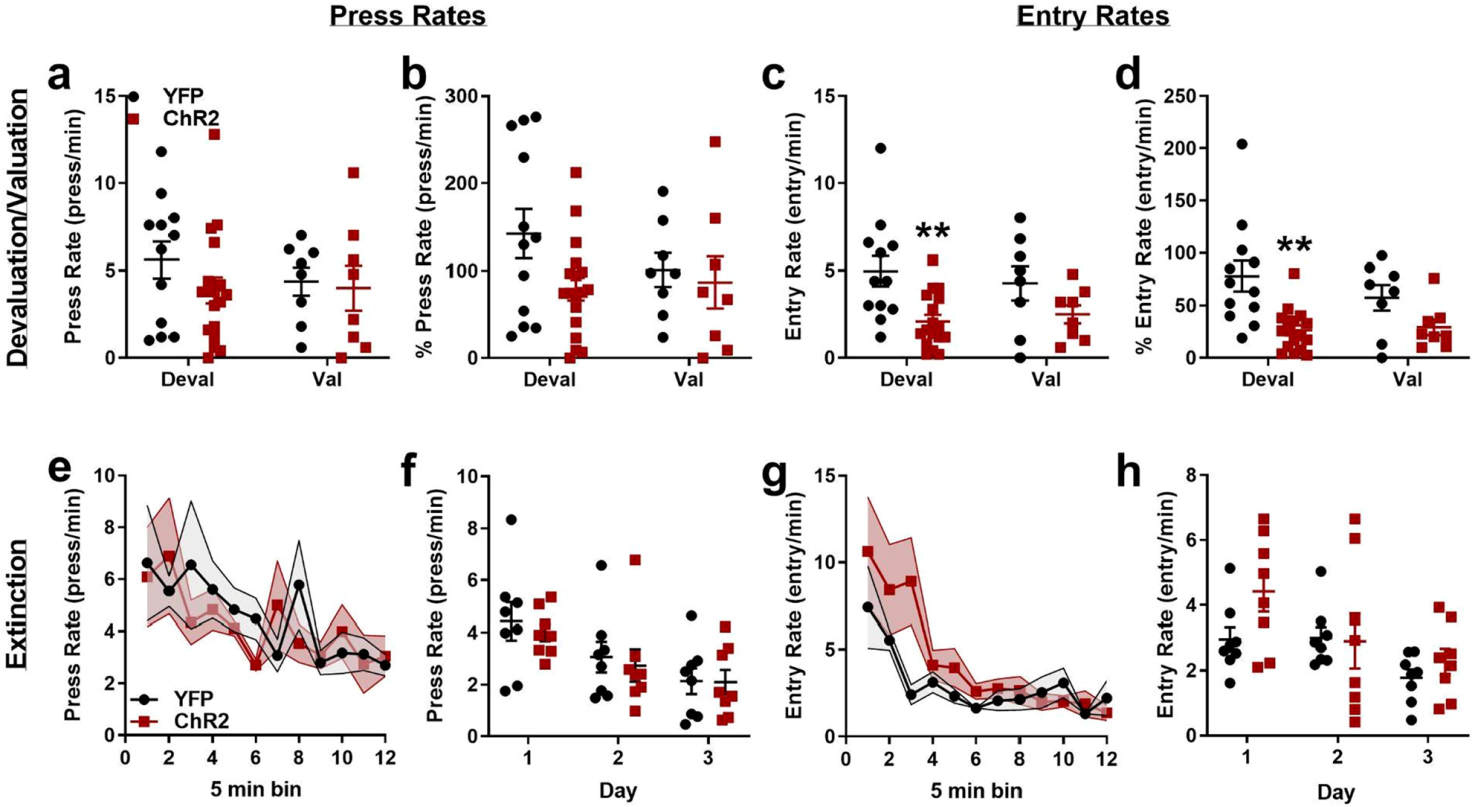
Effects of optogenetic patch manipulation in learning following probe trials and extinction. (**a**) Average press rates during devaluation and valuation probes for ChR2 and YFP mice. (**b**) Press rates normalized to average VI60 response rates for both probe conditions. (**c**) Average entry rates during devaluation and valuation probe trials. (**d**) Entry rates normalized to average VI60 entry rate for both probe conditions. (**e**) Press rates in 5 min bins across the first day of extinction. (**f**) Average press rates across three days of extinction (**g**) Head entry rates in 5 min bins across 60 min extinction trials (**h**). Entry rates and across three days of extinction. *p < 0.05; **p < 0.01; error bars, SEM.

Habits are operationally defined as behaviors resistant to reward-specific outcome devaluation. Therefore, after the completion of eight VI60 training days, two different groups of mice received free access to either 10% sucrose (devaluation condition) or 10% maltodextrin (valuation condition) for one hour before being returned to behavioral chambers for a 5 min probe trial, respectively (see Fig. 2a for Experimental Design)^33^. ChR2 and YFP mice did not differ in the amount of sucrose or maltodextrin consumed during prefeeding (Supplemental Fig. 3; *p* ≥ 0.169) and during probe trials, lever presses and head entries were recorded, but no rewards were delivered. ChR2 and YFP mice did not significantly differ in press rates during devaluation or valuation probes (two-way ANOVA, no significant effect of stimulation group, probe condition, or interaction, all *p* ≥ 0.3069; Fig. 3a), suggesting both groups were generally habitual in responding. Similarly, when normalized to average response rates in VI60, neither ChR2 nor YFP mice showed significant differences across devaluation and valuation probes (two-way ANOVA, no significant effect of probe condition, F_(1,42)_ = 0.551, *p* = 0.462, no significant group x probe interaction F_(1, 42)_ = 1.127, *p* = 0.294), again reflecting intact habits. However, stimulation of patches during training may have modified responding in probe trials: ChR2 mice had slightly lower press rates relative to YFP mice (effect of stimulation group, F_(1,42)_ = 2.848, *p* = 0.099; Fig. 3b).

Mice also had similar head entry rates across devaluation and valuation probes (two-way ANOVA, no significant effect of probe type, F_(1, 41)_ = 0.04179, *p* = 0.8390; no significant probe x group interaction, F_(1, 41)_ = 0.6164, *p* = 0.4369), which again suggests habitual responding in both YFP and ChR2 mice. However, ChR2 mice entered the reward port less frequently than YFP controls during probe trials (significant effect of stimulation group, F_(1,41)_ = 11.05, *p* = 0.0019). Sidak’s post hoc tests indicated that this main effect was driven by lower entry rate in the devaluation condition (*p* = 0.0029) but not valuation condition (*p* = 0.2246) (Fig. 3c). When entry rates were normalized to average VI60 rates, mice also had no difference between probe types (two-way ANOVA, no significant effect of probe type, F_(1, 41)_ = 0.6914, *p* = 0.4105; no significant probe x group interaction, F_(1, 41)_ = 1.249, *p* = 0.2702). Normalized entry rates were also decreased in ChR2 mice (significant effect of stimulation group, F_(1,41)_ = 14.57, *p* = 0.0004), with Sidak’s post hoc tests again revealing this effect being driven by lower normalized entry rates in the devaluation (*p* = 0.0004) versus the valuation condition (*p* = 0.1874) (Fig. 3d). Thus, while both ChR2 and YFP groups displayed habitual responding across devaluation and valuation probes, ChR2 stimulation decreased both press and entry rates across probe trials, specifically in the devaluation condition. This finding could suggest two possibilities: 1. that extinction rates are increased following patch stimulation, and 2. that habit formation may be subtly modified considering the extinction is specific to devaluation rather than valuation conditions.

In addition to habit formation, the striatum is also deeply involved in extinction of previously learned motor behaviors^34^. Indeed, across all probe conditions a general tendency for decreased responding is noted in ChR2 mice, suggesting that ChR2 manipulations may generally modify extinction rates. To directly test this, a subset of mice were retrained on the VI60 schedule for one day following probe trials before beginning three days of extinction, which lasted a total of 60 min each day. Here, levers were extended and mice could freely respond, but pressing resulted in no rewards. ChR2 and YFP mice did not differently extinguish responding based on press rates within the first extinction session (two-way repeated measures ANOVA, no significant effect of group or interaction, both *p* > 0.7132, near-significant effect of time F_(3.849, 50.04)_ = 2.508, *p* = 0.0556; Fig. 3e) or across three days of extinction (two-way repeated measures ANOVA, no significant effect of group or interaction, both *p* > 0.6922, significant effect of time F_(1.362, 19.07)_ = 17.24, *p* = 0.0002; Fig. 3f). In contrast, ChR2 mice had slightly higher head entry rates early in the first day of extinction (two-way repeated measures ANOVA, trending effect of group, *p* = 0.073, trending interaction, *p* = 0.0987, significant effect of time F_(2.731,35.50)_ = 8.064, *p* < 0.0005; Fig. 3g), though entry rates did not differ across three days of extinction trials (Two-way repeated measures ANOVA, no significant effect of group or interaction, *p* = 0.1679, significant effect of time F_(1.865, 26.11)_ = 8.45, *p* = 0.0018; Fig. 3h). Thus, ChR2 activation of striatal patches does not seem to enhance extinction of lever pressing, though it may partially slow extinction of reward-seeking by driving a small “extinction burst”^35,36^, which may further suggest goal-directed responding (see “Discussion” section). This data, taken together with the results of devaluation and valuation probes, suggests that modifying patch activity during training may modify responding during devaluation probes in a manner that cannot be solely attributed to changes in extinction rates, indicating modified habit formation.

One day after the probe test, mice that underwent devaluation were retrained on a VI60 schedule with optogenetic stimulation of patches to reinstate robust pressing before beginning two days of omission probes (see experimental design Fig. 2a). In omission, mice were required to abstain from pressing for 20 s in order to receive a reward. This probe has been used as an alternative means to assess habit by measuring flexibility in forming new action-outcome contingencies^37,38^, and we previously reported that mice with patch lesions have reduced pressing in omission trials. However, we did not observe any effect of ChR2 stimulation on raw or normalized press or entry rates relative to controls (all *p* ≥ 0.2130 with the exception of normalized entry rates, significant group × time interaction *p* = 0.0138; no significant post hoc tests, *p* ≥ 0.674; Supplemental Fig. 1). As expected, YFP mice had a strong correlation between press rate on day 1 of omission and press rate on the reinstatement day between devaluation and omission probes (Pearson’s correlation, R^2^ = 0.551, *p* = 0.0057; Supplemental Fig. 1). However, ChR2 mice did not display any correlation between press rates during these days (R^2^ = 0.0095, *p* = 0.6993; Supplemental Fig. 1). This finding could further suggest impaired day-to-day consistency in responding in patch stimulated mice.

### Determining patch contributions to locomotion and place preference

**Figure 4:**
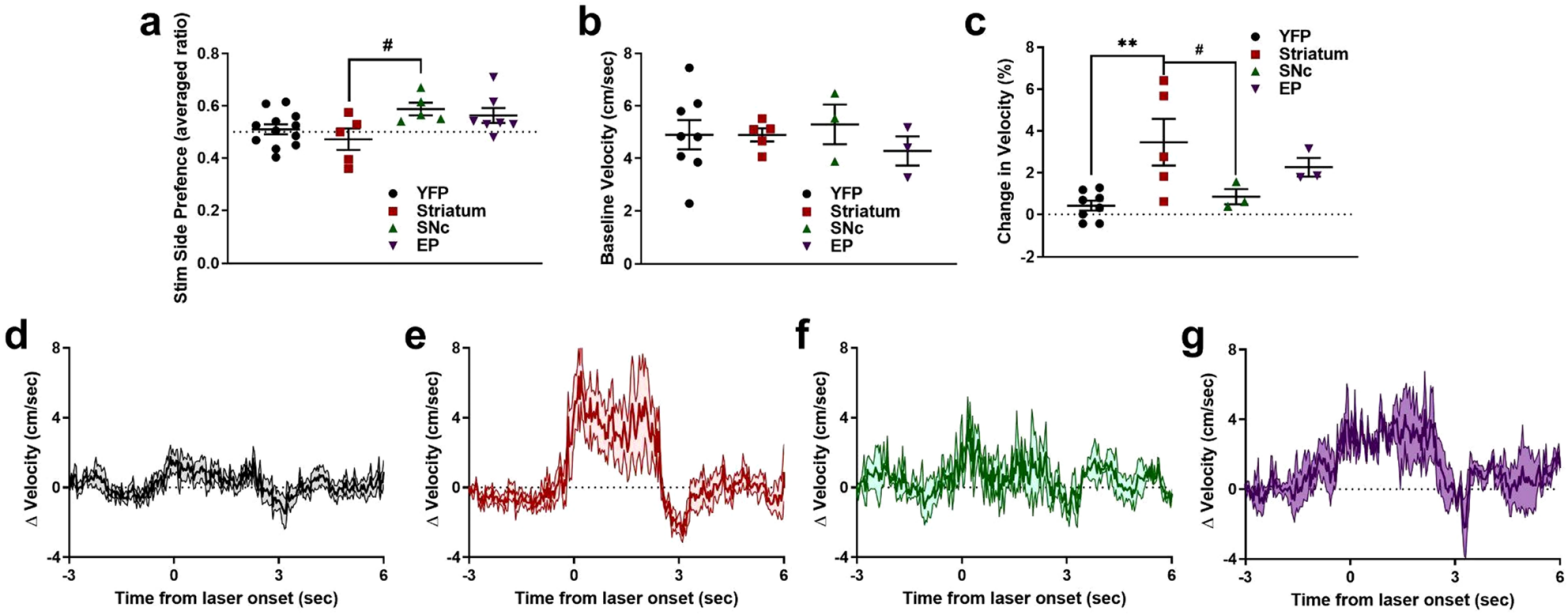
Effects of optogenetic stimulation of patches on reinforcement and locomotion. (**a**) Time spent on stimulated side of a two-chamber place preference apparatus. Time is averaged between two days, and the stimulated side was counterbalanced between days (see Methods). (**b**) Average velocity in the open field for YFP and ChR2 groups. (**c**) Change in velocity following laser onset in open field. (**d**–**g**) Average baseline normalized velocity before and after laser onset (5 Hz, 3 s; denoted by thick black line) for YFP (**d**), striatum (**e**), SNc (**f**), and EP (**g**) stimulation site groups. *p < 0.05; ^#^*p* < 0.1; error bars, SEM.

Patch stimulation could modify responding in the VI60 task by being inherently rewarding^19^. To explore this possibility, we next investigated the effects of patch stimulation on reinforcement in a place preference task. To test the effects of optogenetic stimulation on reinforcement, mice began a two-day, real-time place preference task in a 2-chamber apparatus. Entry to a randomly selected side resulted in laser stimulation (5 s ON, 5 mW, 5 Hz; and 5 s OFF, cycled), which ended upon entrance to the opposite chamber. The stimulation side was counterbalanced across 2 days and preference was averaged between days (see Methods). Patch activation did differentially affect responding across subgroups (one-way ANOVA, F_(3,25)_ = 3.023, *p* = 0.0484, Fig. 4a), and post hoc tests indicated this difference was driven by a trending difference between Striatum and SNc groups (Tukey’s test, *p* = 0.0729, all others *p* > 0.10). These results suggest that optogenetic stimulation of patches is not inherently reinforcing in this place preference task, though subtle differences may be present between stimulation sites.

Due to a predominant D1 dopamine receptor makeup^28^, patch stimulation may alter behavior by invigorating movement^39^. To explore this possibility, mice were placed in an open field chamber to assess the effects of patch stimulation on locomotion, and laser stimulation (3 s, 5 mW, 5 Hz) occurred every 60 s while the location of the mouse was tracked (see Methods). We first assessed if intermittent patch stimulation elevated average locomotion throughout the task, and found no difference in movement speed between ChR2 and YFP mice, regardless of implant group (one-way ANOVA, F_(3,15)_ = 0.3312, *p* = 0.8029, Fig. 4b). However, onset of laser stimulation differentially affected implant groups (one-way ANOVA, F_(3,15)_ = 5.221, *p* = 0.0115). *Post-hoc* tests indicated this difference was due to elevated locomotion in striatum-stimulated group relative to YFP mice (Tukey’s test, YFP vs. striatum: 0.0054) and subtle differences between striatum-stimulated and SNc mice (Tukey’s test, YFP vs. striatum: 0.096, all other comparisons *p* > 0.05). On a finer timescale, striatum-stimulated mice demonstrated robust initial increases following laser onset, which plateaued until cessation of stimulation, followed by a short reduction in movement (Fig. 4e). In contrast, SNc-stimulated mice demonstrate little increase in movement (Fig. 4f), and EP-stimulated mice demonstrated moderate (but non-significant) activation (Fig. 4g). YFP controls showed only modest responses to laser stimulation (Fig. 4d). Together, these results show that patch activation can acutely drive locomotion without being intrinsically reinforcing.

### Characterization of patch and dopamine interactions in vivo

**Figure 5:**
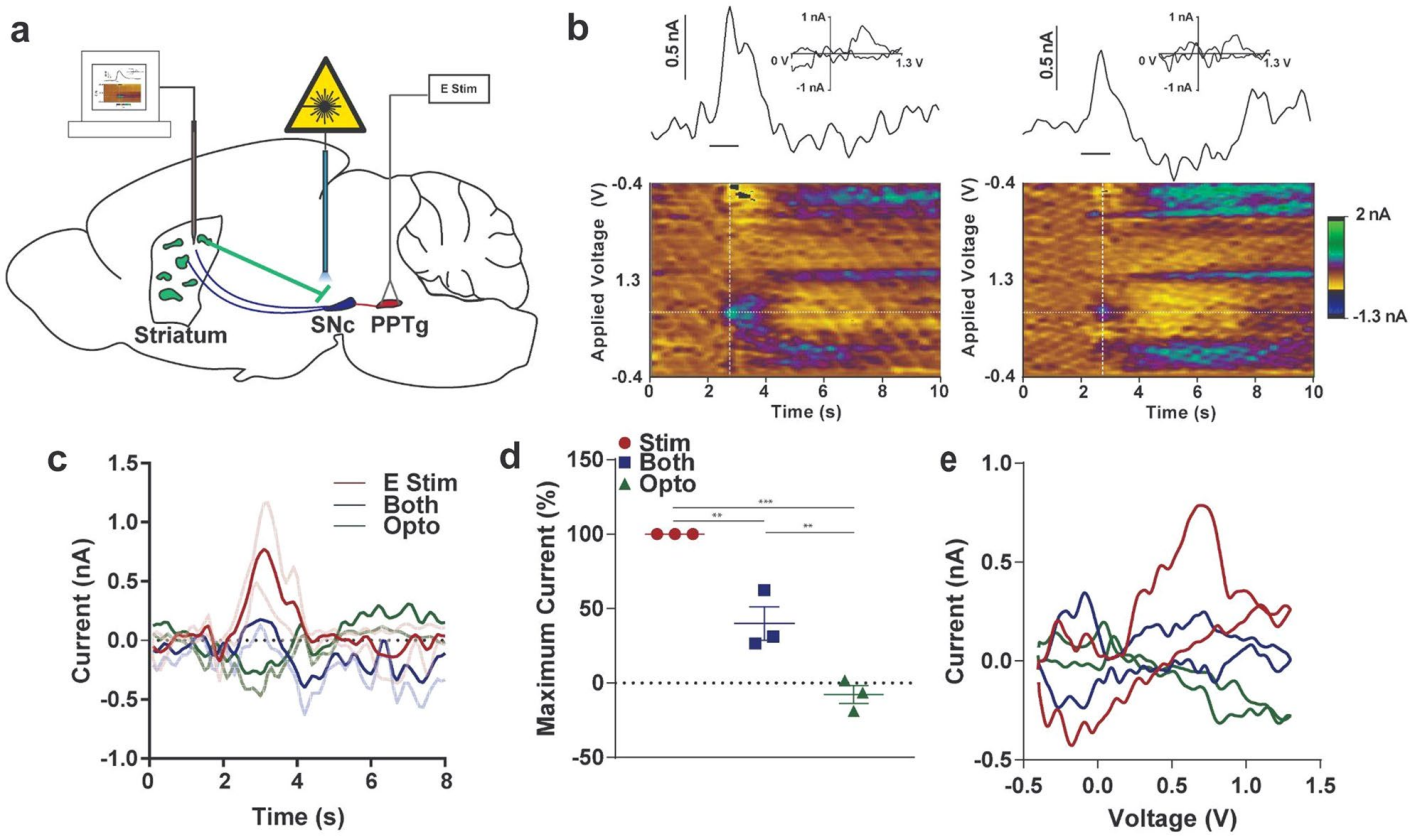
Characterizing optogenetic patch stimulation on striatal dopamine release. (**a**) Experimental design. Sepw1 NP67 mice were injected with AAV5 driving Cre-dependent expression of ChR2-eYFP. Fast-scan cyclic voltammetry was used to monitor real-time changes in dopamine levels in striatum. An electrical stimulating electrode was placed in the pedunculopontine nucleus (PPTg) to drive increases in dopamine in the dorsal striatum. A fiber optic was placed above patch terminals in the substantia nigra pars compacta (SNc). Electrical or optogenetic or both stimulation types were randomly selected and delivered a minimum of three times each (see Methods). (**b**) (left) Representative recording of electrical stimulation of PPTg. Here, a line shows recorded current relative to stimulation delivery (straight line below current trace) above a pseudo-color plot. The color plot shows current collected (in color) at each waveform scan (y-axis) and across time (x-axis). INSET: a “cyclic voltammogram” collected at the vertical white dotted line on the pseudo-color plot suggesting that dopamine is the predominant analyte being monitored. (right) Same as left, but optogenetic stimulation of patch terminals occurs simultaneously with PPTg electrical stimulation. (**c**) Average responses across replicates for each of the three stimulation types. PPTg stimulation only denoted as E Stim, optogenetic stimulation of patch terminals as Opto. (**d**) Maximum recorded current during stimulation normalized to electrical PPTg stimulation. (**e**) Average cyclic voltammogram across replicates for each stimulation type. *p < 0.05; **p < 0.01; ***p < 0.001; error bars, SEM. (**a**) drawn in Illustrator CC 2019 (Adobe).

As striatal patches are a unique striatal output population projecting to SNc^16,40,41^, optogenetic stimulation of patches may alter instrumental learning by modulating dopamine release. We aimed to investigate this possibility by eliciting phasic increases in striatal dopamine with electrical stimulation of the pedunculopontine tegmental nucleus (PPTg)^42^ with and without simultaneous optogenetic activation of patch projections to the SNc. We first injected a group of Sepw1-NP67 mice with an AAV encoding Cre-dependent ChR2 in the striatum. Three weeks later, mice were anesthetized and a glass-sealed carbon-fiber microelectrode capable of detecting real-time changes in dopamine with fast-scan cyclic voltammetry was lowered into the dorsal striatum. Following this, a bipolar stimulating electrode was placed in the PPTg. Once robust dopamine was detected, a fiber optic was lowered into the SNc targeting patch terminals (see Fig. 5a for experimental design).

Stimulation of PPTg resulted in increases in striatal dopamine that mimicked naturally occurring phasic increases in dopamine^43,44^ (Fig. 5b, left). When PPTg stimulation occurred simultaneously with optogenetic patch activation, phasic dopamine responses were present, but reduced in amplitude (Fig. 5b, right). On average, PPTg stimulation alone resulted in larger responses relative to simultaneous electrical and optogenetic stimulation. On the other hand, optogenetic patch activation alone caused a small decrease in detected current (Fig. 5c; Supplemental Fig. 4a) followed occasionally by an increase in current that could represent rebound activity in dopamine neurons (Fig. 5c, Supplemental Fig. 4a and b)^16^. When recordings were normalized to average PPTg stimulation recording amplitude to account for baseline differences in release between subjects, PPTg stimulation drove a larger dopamine response than simultaneous PPTg and patch activation, which was significantly higher than optogenetic activation alone (one-way ANOVA, F_(2,6)_ = 53.97, *p* < 0.0001; post hoc Tukey multiple comparison test all *p* < 0.01; Fig. 5d). Inspection of average cyclic voltammograms further suggests the analyte detected following PPTg stimulation is dopamine, and the peak oxidation is reduced following simultaneous optogenetic and electrical stimulation (Fig. 5e; average current and cyclic voltammograms for each experimental replicate shown in Supplemental Fig. 4). These results suggest that patch projections to dopamine neurons are capable of suppressing dopamine release in the dorsal striatum, which may contribute to the effects noted in the behavioral tasks.

## Discussion

The striatum is a key locus in the transition from flexible to habitual strategies, but less is known about how particular striatal subcircuits contribute to this phenomenon. Previous studies have implicated striatal patches (striosomes) in this process^44–47^, and more recent work has demonstrated that intact patches are necessary for normal habit formation^24,25^. The current work further addresses the role of patches in habit using optogenetics to modulate patch neuron activity in a temporally-precise manner during habit formation. Patch stimulation at reward delivery enhanced behavioral variability and invigorated responding across training by modifying the pattern of responding. Additionally, optogenetic stimulation of patch activity during learning suppressed the rate of lever pressing and head entry during probe trials, with larger decreases following reinforcer devaluation compared to valuation. Moreover, this was not solely attributable to increased extinction rates based on a subsequent extinction probe, suggesting patch stimulation modified habit formation. These effects were not directly attributable to directly rewarding effects of patch stimulation as assessed in a place preference task, though stimulation of patches was sufficient to acutely increase locomotion in an open field. Finally, we demonstrated that optogenetic stimulation of patch terminals is sufficient to suppress dopamine release driven by electrical stimulation of excitatory inputs to dopamine neurons. Together, these results suggest that patches are involved in habit formation and that patch activation may modify habitual responding, potentially through regulation of striatal dopamine levels.

### Patch optogenetic stimulation modifies habit formation

Here, we note reduced responding in devaluation probe trials following ChR2 stimulation of striatal patches in learning, which may suggest modified habit formation (Fig. 3c,d). It is important to note that we did not find specific differences in devaluation vs. valuation probe conditions, a traditional characteristic of goal-directed behavior^3,8,27^. Instead, here, we note partial decreases in head entry rates across both probe conditions that are largely attributed to decreased responding by ChR2 mice in devaluation. In support of this, subsequent extinction experiments (Fig. 3e–h) demonstrated that optogenetic stimulation of patches is not sufficient to reduce responding in extinction, suggesting effects in devaluation were not due to generalized elevation in extinction rates. Further, we noted an unexpected increase in head entries early in the first extinction session, which may reflect an “extinction/response burst”^35,36^ or “frustration effect”^48^, where animals increase responding following omission of expected reinforcers, though this effect is only trending toward statistical significance here. Interestingly, these extinction bursts are typically only noted in animals trained using continuous reinforcement (FR1)^36^ or variable ratio schedules^35^ as opposed to variable interval schedules^49^, which is notable due to the well-documented ability of these tasks to facilitate goal-directed or habitual responding, respectively^4,8,50^. Thus, the subtle response burst noted here (Fig. 3g) may provide ancillary evidence of goal-directed responding in mice that received optogenetic stimulation.

In support of this conclusion, striatal patches have been shown to be necessary for habitual responding by previous studies using Cre-dependent caspase lesions^25^ or a conjugated cytotoxin that selectively ablates µ-opioid receptor expressing neurons^24^. These patch manipulations, and the optogenetic approach utilized here, target the central striatum, likely affecting patches in both medial and lateral striatum. A well-supported model proposes that the medial striatum supports goal-oriented behavior, while the lateral striatum supports habitual responding^5^, a distinction which may also apply to dopamine neurons^50–53^. Patches span the anterior/posterior and medial/lateral spectrum of the striatum^54^, and studies manipulating patches specifically within different areas of the striatum should be pursued to determine if there is a functional divide within medial and lateral patches. Notably, expression of key neurochemical patch markers differs across striatal subregions, suggesting potential heterogeneity of patch function across the striatum^55^.

One puzzling aspect of the current work is how optogenetic stimulation of patches could impair responding following reinforcer devaluation, a finding similar to previous lesion studies, when one may anticipate directly opposing effects of lesion and optogenetic stimulation manipulations. We propose four possible explanations to this paradoxical finding. First, optogenetic stimulation could disrupt the timing of spiking relative to afferent activation during learning, thus interfering with spike-timing plasticity necessary for the transition to habitual responding. Striatal neurons have been shown to be highly sensitive to spike-timing of corticostriatal inputs, and modifying afferent and spiny projection neuron spike timing by milliseconds can reverse the valence of plasticity in these cells^56^. We chose to deliver optogenetic stimulation during reward retrieval based on previous studies showing increases in activity in patches to rewards or cues predicting rewards^18^. However, direct electrophysiological or optical recordings of patches are required to assess how our optogenetic manipulation modified ongoing activity.

Second, optogenetic activation of patches may drive rebound inhibition, which could lead to impairments in habit formation by suppressing patch activity following cessation of laser firing. Rebound excitation and inhibition have been well characterized during inhibition or excitation of neuronal circuits with optogenetics^57^. Indeed, we note potential behavioral evidence of this phenomenon in the current work: patch activation in open field drives robust increase in locomotion, followed by a brief inhibition of movement where locomotor behaviors fall below baseline (Fig. 4d–g).

Third, the selection of 5 Hz optogenetic stimulation may have contributed to the similarities between optogenetic manipulations and previous lesion studies^24,25^. Striatal projection neurons tend to fire below 5 Hz in awake, freely behaving rats^58,59^, and patches have been similarly reported to fire below ~ 3 Hz^21^. However, patch neurons burst fire to salient stimuli^18,20^, and striatal projection neurons have been shown to fire at frequencies above 10 Hz to salient events^59^. While it is not known what firing frequencies patch neurons achieve during variable interval responding, recordings ex vivo have demonstrated that patch neurons are capable of achieving firing frequencies as high as 45 Hz following current injection^15^. Therefore, our 5 Hz stimulation delivered at reward retrieval may enhance baseline activity of patch neurons, or could alternatively suppress reward-related burst activity. Low-frequency optogenetics of excitatory neurons has been shown to reduce high-frequency, ictal activity, demonstrating that it is possible to disrupt ongoing bursting activity with low-frequency stimulation of ChR2^60,61^. It is therefore possible that higher stimulation frequencies (15 Hz, for example) could reverse the effects on habit formation noted here by augmenting activity associated with reward retrieval. The 5 Hz stimulation frequency utilized here was selected due to the robust effect on locomotor activity noted in the open field, and we reasoned that higher stimulation frequencies may have disrupted operant responding under a variable interval schedule.

Finally, it is possible that timing the delivery of optogenetic stimulation specifically to reward retrieval contributed to impaired habit formation and that modifying the timing of stimulation or the valence of the associated event may alter this effect. While models have arisen characterizing patches as a reward-evaluation element within the striatum^62,63^, alternative models posit that patches bias matrix neurons to guide action selection^64^. Additionally, striatal neurons encode the initiations, terminations, and transitions between elements of operant behaviors^65,66^, though their compartmental origin had not been identified. Therefore, modifying patch activity at alternative points in variable responding, such as during lever pressing, could differentially modify operant learning and habit formation. Future studies are needed to characterize patch activity during variable interval responding, and experiments using optogenetics to modify patch activity in habit-forming tasks could employ a range of stimulation frequencies and stimulation timing to explore these possibilities.

A limitation of the current work is the lack of a within-subjects control for reinforcer-specific changes in valuation during our assessment of habit formation. Devaluation and valuation probes are often performed in the same group of animals, counterbalanced across days^8,27^. Here, devaluation probes and valuation probes were performed in separate groups of mice^4^. Further, these separate groups had different numbers of mice and asymmetric fiber optic placement subgroups, resulting in an imbalanced comparison. Separate valuation and devaluation groups were used to account for our previous finding that Sepw1-NP67 mice rapidly suppress responding across two days of probe trials regardless of reward type, which obscured results of devaluation in our previous lesion study^25^. Therefore, while effects of optogenetic stimulation of patches is statistically specific to the devaluation condition which suggests modified habit formation, some caution should be exercised in this interpretation.

### Optogenetic manipulation of patches modifies behavioral variability

During training, optogenetic stimulation of patches resulted in lower autocorrelation coefficients (Fig. 2e and i), suggesting impaired day-to-day consistency in responding. Further, correlation of responding during retraining and omission probes were disrupted in ChR2 mice (Supplemental Fig. 1g, h which may reflect enhanced behavioral variability across days. These findings are supported by our previous study, which found that Cre-dependent lesions in Sepw1-NP67 mice similarly disrupted autocorrelations and increased behavioral variability^25^. These studies together suggest that patches may support habit formation by facilitating crystallization of action patterns. In support of this notion, optogenetic manipulation of patches altered the pattern of responding, but not the speed of transitions, during VI60 training (Fig. 2j–m). Moreover, generalized lesions of the dorsal striatum have been shown to increase behavioral variability in foraging tasks^67^. Together, these studies suggest patches contribute to stabilizing behavior across learning.

### Differential contributions of divergent patch outputs to behavior

A surprising finding in the current work was that optogenetic activation of patch projections to EP, SNc or patch neurons in the striatum did not differentially modify responding in VI60, devaluation probes, or omission (Supplemental Figs. 1–3). Spiny projection neurons (SPNs) in both striatal patches and in the surrounding matrix project to canonical basal ganglia targets in the pallidus and substantia nigra, but with some key differences^12,17,68^. Patch SPNs, compared to matrix SPNs, provide stronger functional output from the striatum to dopamine neurons in the SNc^16^ and preferentially innervate glutamatergic (or GABA/glutamate co-releasing) habenula-projecting EP neurons^30^. These EP neurons, in turn, project to the lateral habenula (LHb) and may influence dopamine signaling via the rostromedial tegmental nucleus, a major output nucleus of the LHb that strongly inhibits dopamine neurons^67–71^. However, detailed anatomical tracing indicates that most patch SPNs either send collaterals to both SN and EP, or exclusively project to the GPe^68^. Thus, the patch projections we stimulated (to the SN and EP) may not be opposing “direct” and “indirect” projections, and may be expected to play similar roles in behavior. On the other hand, despite no changes between implantation sites in VI60 and in habit probes, we did note differences in place preference and open field. Across these tasks, EP stimulation was more, and SNc stimulation was less, behaviorally-activating (Fig. 4), which aligns with their putative dopamine-eliciting or dopamine-inhibiting downstream circuits, respectively. Further anatomical and functional data are needed to firmly establish projection-specific roles patch SPNs play in various behaviors, as these collaterals to the SN and EP may still play different roles in behavior despite originating from the same neurons, as indicated by some implantation site differences observed in this work. Future studies should also examine the impact of patch to EP stimulation on striatal dopamine release.

### Patch control over dopamine release

This work provides new insight into the relationship between striatal patches and dopamine release, demonstrating that optogenetic stimulation of patch projections suppresses dopamine release in the dorsal striatum (Fig. 5). Previous studies have supported this notion demonstrating anatomical and functional connectivity between patch projections and dopamine neurons in the SNc, primarily through striosome-dendron bouquets that descend into the SNr^40,41^. Patches could therefore modify learning by directly inhibiting dopamine neurons, or by shifting the timing of dopamine signals. Indeed, dopamine responses transition from ventromedial to dorsolateral striatum as drug-seeking behaviors become well learned^72^, and patches could be involved in gating of dopamine release early in learning. Very recent work suggests that patch-dopamine interactions are also reciprocal, as dopamine differentially modulates patch neuron activity relative to matrix neurons^73^.

One paradoxical finding of the current work is that patch stimulation invigorates responding in VI60 and enhances locomotion, while also inhibiting dopamine release in our anesthetized preparation. Previous work in rodents has linked increases in dopamine to increased locomotion^74,75^, which is consistent with canonical basal ganglia direct/indirect pathway function, and which seems to directly conflict with our result. The simplest explanation is that we utilized different optogenetic stimulation patterns in behavioral and physiological experiments. In the voltammetry experiments, we used laser stimulation with longer pulse duration and often higher power than that in the open field and other behavioral experiments. However, both stimulation patterns likely result in net activation of patch output; therefore, we propose two alternative potential mechanisms to reconcile these disparate findings: First, patches may be composed of distinct populations of neurons, with subpopulations preferentially enhancing behaviors or suppressing dopamine, respectively. Patch activation of behavior may be expected due to the abundance of D1-expressing neurons in patches^28^, as optogenetic stimulation of D1 neurons enhances movement through inhibition of basal ganglia output nuclei^76^. Although patch neurons project to the SNc, they also project to the SNr, a canonical basal ganglia output nucleus directly ventral to the SNc^17^. Anatomical evidence suggests that individual patch neurons project to both the SNc and the SNr^68^. Thus, it is entirely possible that some of our laser stimulation affected patch axons in the SNr. As activation of the direct pathway projection to the SNr is behaviorally invigorating^39,76^, this may be a source of the invigoration we observed. Furthermore, a recent study suggests that striosomes can be further subdivided into functionally distinct populations, both of which predominantly express D1 receptors. Optogenetic stimulation of Teashirt family zinc finger 1 (Tshz1) expressing neurons in striosomes drives aversion and suppression of movement^77^, which is consistent with suppression of dopamine^69,78^. On the other hand, optogenetic activation of prodynorphin expressing neurons, which are also enriched in patches and which have also been shown to acutely suppress dopamine release^16^, paradoxically drives reinforcement^77^. Therefore, Sepw1 NP67 mice may encompass a mixed population of these neurons and/or other currently-uncharacterized subpopulations of patch neurons that preferentially drive locomotion and suppress dopamine, respectively.

Alternatively, patch modulation of dopamine levels may be more dynamic than our results first suggest. Patches have been shown to drive rebound activity in dopamine neurons following initial inhibition^16^, which is consistent with pause-rebound dopamine activity following aversive stimuli^52^. This activity is consistent with the tendency of dopamine traces to recover following initial decreases in our “laser only” trials shown here. However, the potential rebound signals detected here are small, and they lie just at the threshold for detection by FSCV (Fig. 5c, Supplemental Fig. 4a and b). Nevertheless, these studies together suggest patch neurons may dynamically modulate, rather than completely suppress, dopamine release. Similarly, we are unable to distinguish dopamine concentrations across patch/matrix boundaries. It is possible dopamine release is differently regulated across patch/matrix boundaries, which has been previously demonstrated^77–81^. It would be of great interest to detect dopamine levels across patches and matrix simultaneously with patch neuron activity in awake behaving animals to determine how these systems interact during behavior.

## Conclusion

In sum, the current work suggests that activation of striatal patches invigorates ongoing behaviors, which was noted in instrumental responding during VI60 as well as locomotion in the open field. Patch stimulation during training facilitated goal-directed responding during devaluation trials and a small response burst in extinction, which may reflect goal-directed responding. These findings were not attributable to large differences in stimulation preference across implantation site groups, though divergent stimulation sites differently modified locomotion increases. Surprisingly, behavioral activation noted here was accompanied by decreases in striatal dopamine, which could reflect different regulation of dopamine across patch matrix boundaries, or direct activation of D1 terminals in SNc. Many questions remain regarding the role of patches in habit formation:

1. What is the pattern of patch neuron activity during variable interval responding, and how does it change across habit formation?
2. How might altering stimulation timing and frequency affect the current results? In particular, how does enhancing/inhibiting activity at reward retrieval differ from modifying activity at other points in the task (i.e., lever pressing)?
3. How does patch and dopamine activity co-fluctuate in behaving animals, and how exactly do patches simultaneously drive behaviors and suppress dopamine release?
4. How exactly do differential patch outputs contribute to complex behavioral regulation?
5. Given the interaction of patch/dopamine systems, what role do patches play in pathological compulsive states including drug addiction and Obsessive Compulsive Disorder?

## Materials and methods

### Animals

All experiments were approved by the Oberlin College Institutional Animal Care and Use Committee and were conducted in accordance with the National Institutes of Health’s Guide for the Care and Use of Laboratory Animals and Animal Research: Reporting of In Vivo Experiments (ARRIVE) guidelines. Mice were maintained on a 12 h/12 h light/dark cycle and unless otherwise noted, were provided ad libitum access to water and food. Experiments were carried out during the light cycle using 49 heterozygous Sepw1-Cre^+/−^ mice ranging from 2 to 6 months of age, which were generously provided by Charles Gerfen (National Institutes of Health) and Nathanial Heintz (Rockefeller University). These mice preferentially express Cre-recombinase in striatal patches^26,28^ (Fig. 1c + d).

### Reagents

Isoflurane anesthesia was obtained from Patterson Veterinary (Greeley, CO, USA). Sterile and filtered phosphate buffered saline (PBS, 1X) was obtained from GE Life Sciences (Pittsburgh, PA, USA). Unless otherwise noted, all other reagents were obtained through VWR (Radnor, PA, USA).

### Stereotaxic surgery and viral injections

Sepw1-NP67 mice were anaesthetized with isoflurane (4% at 2 L/s O2 for induction, 0.5–1.5% at 0.5 L/s O2 afterward) and then placed in a stereotactic frame (David Kopf Instruments, Tajunga, CA, USA). The scalp was sterilized with povidone iodine and an incision was made in the scalp. For optogenetic experiments, the skull was scored with Optibond (Patterson Dental). Holes were then drilled bilaterally above the dorsal striatum (+ 0.9 AP, 1.8 ML, − 2.5 DV) and 500 nL of an AAV encoding channelrhodopsin (ChR2) (AAV-EF1-DIO-hChR2(H134R)-EYFP-WPRE-pA, UNC Viral Vector Core) was injected. Control mice were injected with an AAV encoding YFP (AAV-EF1a-DIO-EYFP, UNC Viral Vector Core). For all injections, a 5 µL syringe needle (Hamilton) was lowered to the DV coordinate over 2 min and held in place for 1 min before the start of injection. The injection speed was 100 nL/min, and the needle was left undisturbed in the brain for 5 min after the completion of virus delivery, after which the needle was removed over the course of 5 min. Fiber optics were then inserted bilaterally targeting one of three sites: cell bodies of patch neurons in the striatum (+ 0.9 AP, 1.8 ML, − 2.3 DV), patch terminals at dopamine neurons of the substantia nigra pars compacta (− 3.2 AP, 1.5 ML, − 3.6 DV), or over patch terminals in the entopeduncular nucleus EP (− 1.1 AP, 2.1 ML, − 4.0 DV; Fig. 1a + b), and secured to the skull with dental cement (Patterson Dental). Control mice expressing YFP had fiber optics implanted targeting one of these three sites selected randomly. Carprofen (5 mg/kg, subcutaneous) was used for postoperative analgesia. A subset of mice were injected with AAV encoding ChR2 but did not receive fiber optic implants. These mice instead received sterile sutures to close the incision site (see Fast-Scan Cyclic Voltammetry below). All mice were given 3–4 weeks to allow for viral expression and to recover before behavioral training started.

### Variable interval training

Mice were trained on a variable interval schedule to induce habitual responding^31^ (see Fig. 2a for schematic of entire behavioral training protocol). Throughout training, mice were food deprived and kept at ~ 85% initial weight by daily feeding of 1.5–2.5 g of homecage chow daily after training. All instrumental learning experiments were performed in standard operant chambers (Med Associates). Each chamber had a retractable lever on either side of a reward bowl, which was linked to a sucrose-filled syringe that delivered liquid reward (10% sucrose solution, 20 µl) and a house light on the opposite side of the chamber. Briefly, mice first underwent four days of continuous reinforcement (CRF, one lever press yields one reward) to establish the association between lever press and reward. At the start of the session, the house light was illuminated, and one lever was inserted into the chamber. After 60 min or 50 rewards, the light was shut off, the lever was retracted, and the session ended. On the final day of CRF training, mice were briefly anesthetized with isoflourane (4%, 2 l/min O2) and were connected to fiber optic leads to habituate mice to the optogenetic apparatus. Mice that failed to reach criteria within four days were given an additional 1–2 days of CRF training. Subsequent behavioral trials began with acute anesthetization with isoflourane and connection to fiber optic leads prior to training. Following CRF training, mice experienced three days of a variable-interval (VI) 30 task, in which they were rewarded on average 30 s (15–45 s range) contingent on lever pressing, followed by 8 days of VI60 training (rewarded every 60 s on average, ranging from 30 to 90 s, with each possible interval separated by 6 s)^25^. VI sessions ended after 60 min or when 50 rewards had been earned. To assess the contribution of patches to habit formation, mice received optogenetic stimulation (5 mW, 5 Hz, 190 ms pulse width, 3 s duration, see below) of patch neurons or terminals during the first headentry following each reward delivery in all VI60 trials. Patch activity is linked to reward-predicting cues or during reward consumption^18,20^, and thus this stimulation timing was selected to modulate ongoing activity in patch neurons. Striatal neurons tend to fire below 5 Hz, but can burst fire at frequencies above 15 Hz^15,21,59^. However, because we detected generalized behavioral activation in VI press and entry rates, and an increase in locomotion in open field at 5 Hz (Figs. 2b–I, 5c–e), we restricted stimulation to 5 Hz to avoid disrupting ongoing behavior in the operant chamber.

### Probe tests

Following 8 days of VI60 training, a reward devaluation or valuation test was conducted. Before devaluation probes, mice were given free access to sucrose to devalue the reinforcer they earn in VI60 trials for for one hour prior to testing. A separate group of mice received access to 10% maltodextrin to serve as a valuation probe to control for general extinction driven by satiety^33^. Mice were individually caged during free access and all mice were observed to ensure they consumed rewards. To quantify consumption, a subgroup of mice had sucrose or maltodextrin bottles weighed before and after free access. After the pre-feeding session, mice were given a 5-min probe test in which the lever was extended and presses were recorded, but no rewards were delivered.

All mice that underwent devaluation then experienced one day of VI60 training to reinstate response rates. The following two days, mice were tested on a 60 min omission probe test in which the action-outcome contingency was reversed. Here, mice had to refrain from pressing the lever for 20 s to obtain a reward, and pressing the lever reset the timer^25^. This probe was employed as a second metric of habitual responding, as habitually responding mice are slower to reverse learned action-outcome contingencies^82^.

All mice that underwent valuation probes were retrained in VI60 for one day to reinstate response rates before beginning three days of extinction. In extinction, mice were placed in operant chambers and connected to optogenetics leads for a total of 60 min, and levers were extended but no rewards were delivered and no laser stimulation took place. Extinction trials were conducted to determine if optogenetics of patch cells modified rates of extinction, in which the striatum is known to be involved^34^.

### Fiber optic implants

Fiber optic implants were custom fabricated and were comprised of 0.39 NA, 200 µm core Multimode Optical Fiber (ThorLabs) inserted into a multimode ceramic zirconia ferrules (1.25 mm OD, 230um ID; SENKO). The fiber optic was affixed in the ferrule with two-part epoxy (353ND; Precision Fiber Products). Each end of the fiber optic was polished using fiber optic sandpaper (ThorLabs) and functionality was tested ensuring minimal loss of light power and even output prior to implantation.

### Laser stimulation

Mice received blue laser stimulation (473 nm, 5 mW, 5 Hz, 190 ms pulse width, 3 s duration) from a diode-pumped single-state laser (Laserglow) which was connected via fiber optic (Doric Lenses) to a commutator (1 × 2 Fiber-optic Rotary Joint), allowing for free rotation and splitting of the beam (Doric Lenses). The commutator was connected to two fiber optic leads, which were attached bilaterally to ferrules on fiber optic implants with a ceramic sleeve (Precision Fiber Products). Laser output was calibrated to 5 mW from the end of fiber optic leads before training each day using an optical power meter (ThorLabs). Laser parameters were the same for all behavioral tasks (VI60, Open Field) with the exception of Real-Time Place Preference, where laser stimulation duration was cycled 5 s ON (5 mW, 5 Hz, 190 ms pulse width), then 5 s OFF (see below) when mice were in the laser-paired chamber.

### Real-time place preference

Following operant conditioning tasks, mice were returned to ad libitum access to homecage chow. At least 3 days later, fiber optic implants were again connected to fiber optic leads and mice were placed in a 2-chamber place preference apparatus (Med Associates). Each chamber was 16.8 cm L × 12.7 cm W × 12.7 H with opaque walls. Chambers were distinguishable based on different flooring (grid vs bars) and different wall coloring (white vs black), and the orientation of the chamber did not change across place preference trials. To allow fiber optic movement and prevent mice from exiting the chamber, a custom, clear plexiglass wall extension (45.7 cm tall, 58.4 cm total height) was placed on the walls above the behavioral apparatus and no lid was utilized. Mice underwent two days of real-time place preference trials. Here, one chamber was randomly selected to trigger laser stimulation when mice entered or remained in the ‘active’ chamber, and the active chamber was counterbalanced across days. Location in the chambers was monitored by 12 evenly-spaced infrared beam breaks located near the floor of the apparatus. At the first occurrence of a beam break on the active side, laser stimulation was delivered to the fiber optic implants (5 mW, 5 Hz, 190 ms pulse width). As striatal stimulation can result in freezing depending on the neuronal population activated^39^, laser stimulation was cycled ON for 5 s and OFF for 5 s. This pattern of stimulation occurred until a beam break occurred in the inactive chamber, when stimulation was halted until the next beam break in the active chamber. Time spent on either side was compared and averaged across each day to account for inherent preferences for either side. This task was performed to determine if optogenetic patch stimulation was inherently reinforcing, as suggested by a previous electrical self-stimulation experiment^19^.

### Open field

At least one day following RTPP trials, fiber optic implants were again connected to fiber optic leads and a subset of mice were placed in an open field apparatus (42 cm wide × 42 cm long × 30 cm tall) to determine the effects of acute patch stimulation on locomotor activity. Every 60 s, laser stimulation (5 mW, 5 Hz, 190 ms pulse width, 3 s duration) was delivered to implants. Mouse locomotion was monitored by a camera and analyzed online using Bonsai software (Open-Ephys). Movement was detected using a contrast-based binary region analysis and extraction of location in the video frame^83^. An early pilot group of mice was not included in this open field analysis, resulting in different numbers of animals across RTCPP and open field data sets.

### Fast-scan cyclic voltammetry

To determine the impact of patch activation on striatal dopamine release, we utilized fast-scan cyclic voltammetry (FSCV) to monitor real-time changes in striatal dopamine levels while simultaneously activating patch terminals with optogenetics in vivo. Fast-scan cyclic voltammetry was performed using custom glass-sealed, carbon-fiber microelectrodes^84^. Recordings were made by applying a triangular waveform (0.4 to 1.3 V and back, 400 V/s) every 100 ms to the exposed tips of carbon-fiber microelectrodes. Voltammetry and stimulus control was performed by a WaveNeuro potentiostat (Pine Research) and was computer-controlled using HDCV software, which was generously provided by the Chemistry Department at UNC^85^. A subset of Sepw1-NP67 mice were injected with AAV driving Cre-dependent expression of ChR2 as described above. At least 3 weeks later, these mice were anesthetized using urethane (1 g/kg i.p. delivered in 2 injections separated by ~ 20 min) and placed in a stereotactic apparatus. An incision was made in the scalp and holes were drilled above the dorsal striatum (+ 0.8 AP, ± 1.5 ML), SNc (− 3.2 AP, ± 1.5 ML), and pedunculopontine tegmental nucleus (PPTg, − 0.68 AP from lambda, ± 0.7 ML). The PPTg sends excitatory projections to dopamine neurons and was targeted with electrical stimulation to elicit dopamine release in the striatum^42^. An Ag/AgCl reference electrode was affixed in the superficial cortex. A carbon-fiber microelectrode was placed in the dorsal striatum (− 2.3 DV), and during implantation the carbon-fiber was cycled at 60 Hz to allow the electrode to equilibrate and switched to 10 Hz ~ 20 min prior to data acquisition. A twisted bipolar stimulating electrode (Plastics One, Roanoke, VA, USA) connected to a DS4 Biphasic Constant Current Stimulus Isolator (Digitimer) was lowered in 0.1-mm increments starting at − 1.5 DV into PPTg until robust dopamine increases were detected in the dorsal striatum. Stimulus trains consisted of 60 biphasic pulses delivered at 60 Hz at a current of 400–600 µA and were synchronized with recordings so that sampling and stimulation did not overlap. Stimulation intensity varied across subjects to elicit robust dopamine release, but was fixed at the beginning of data collection and did not alter thereafter. Once stable dopamine release was detected, a fiber optic cable was inserted above SNc (− 2.0 DV) to target patch terminals and was incrementally lowered to optimize placement (see Fig. 5a for graphic of experimental design). Optogenetic stimulation consisted of 1 s pulses of blue laser light delivered at 5–10 mW. Three trial types were then conducted: 1. electrical stimulation of PPTg alone (“E stim” trials), 2. optogenetic stimulation of patch terminals in SNc (“Opto” trials), or 3. simultaneous electrical stimulation of pedunculopontine tegmental nucleus and optogenetic activation of patch terminals (“Both” trials). The order of trials was selected randomly until one of each trial type had been collected, then this process was repeated a minimum of 3 times. All recordings were separated by at least 3 min to avoid neurotransmitter vesicle depletion.

### Histology and microscopy

At the cessation of all behavioral tests, mice were deeply anesthetized with isoflurane (4%, 2 l/min O2) and transcardially perfused with 0.9% saline and 4% paraformaldehyde (PFA). Brains were removed and allowed to post-fix in 4% PFA at 4 °C for at least 24 h. Brains were then transferred to a 30% sucrose solution and returned to 4 °C for at least 48 h. Brains were sectioned on a freezing microtome into 20 μm sections. A subset of striatal sections from optogenetic experiments were mounted and imaged to determine ChR2 expression. A separate set of sections from Sepw1-NP67 mice were washed 3X in Tris buffered saline (TBS) and blocked in 3% horse serum and 0.25% Triton X-100 prior to antibody staining. Sections were then incubated in a 1:500 dilution of anti-μ-opioid receptor polyclonal rabbit antibody (Immunostar, cat #24216) for 24–48 h at 4 °C. Following staining, the tissue was washed again 2X in TBS and then blocked in TBS with 3% horse serum and 0.25% Triton X-100 while being labeled with a donkey anti-rabbit secondary antibody conjugated to Cyanine 3 (Jackson ImmunoResearch, cat#711-165-152). A separate set of tissue was procured from Sepw1-NP67 mice crossed with a Cre-dependent GFP-reporter line to characterize Cre expression. This tissue was processed as described above, but was incubated in a 1:500 dilution of anti-μ-opioid receptor polyclonal rabbit antibody (Immunostar, cat #24216) and anti-GFP polyclonal guinea pig antibody (Synaptic Systems, cat#132-004) for 24–48 h at 4 °C. This tissue was then washed 2X in TBS before being blocked in TBS with 3% horse serum and 0.25% Triton X-100. The tissue was then labeled with a donkey anti-rabbit secondary antibody conjugated to Cyanine 3 (Jackson ImmunoResearch, cat#711-165-152) and donkey anti-guinea pig secondary antibody conjugated to Alexa Flour 488 (Jackson ImmunoResearch, cat#706-545-148). Tissue was visualized using a Leica DM4000B fluorescent microscope or a Zeiss LSM 880 confocal microscope.

### Data analysis

Mean and normalized press and head entry rates were compared across training and probe trials. As press rates in mice with lesioned patches have been shown to be variable across training days^25^, press and entry rates were normalized to average response rate across all VI60 trials to compare to probe trials. Omission press and entry rates were normalized to the reinstatement VI60 training before omission trials. We expected potentially opposing effects of modulating differing terminal sites, but across VI60 training, devaluation probe trials, omission, open field, and place preference tasks we noted no statistical differences between different fiber optic implantation sites across omission, VI training, and devaluation (Supplemental Figs. 1–3). Therefore, groups were collapsed and comparisons were made between ChR2 mice and YFP controls. Behavioral structure was determined using programs written in Python 3.9. The ratio of time spent in active:inactive chambers was averaged across two days of the place preference task and then averaged across groups. Velocity in the open field was calibrated from megapixels/frame to cm/sec using Matlab software MATLAB (R2018b, Mathworks). Press and entry rates were calculated using Excel (Microsoft). Autocorrelations were determined using custom scripts written in MATLAB (R2018b, Mathworks). Extinction press rates from day 1 were determined for each 5 min bin in MATLAB (2020a, Mathworks). FSCV data was analyzed in HDCV (UNC Chemistry Department). Voltammetric current vs time and current vs. voltage traces were collected and averaged for each trial type within experiments (see above) before being averaged between subjects. Evoked amplitudes were normalized to maximum current in PPTg stimulation only trials (‘E stim’ trials) to account for different amplitudes of dopamine responses across subjects. Images in Figs. 1a and b, 2a and 5a were drawn in Illustrator CC 2019 (Adobe).

### Statistical analysis

Statistical analysis was performed by GraphPad Prism 7.04 (GraphPad) or Matlab (R2018b, Mathworks). Press and entry rates during VI60 and omission probes were compared using Two-Way Repeated Measures ANOVA with Geisser-Greenhouse corrections, and, when appropriate, followed by post hoc Sidak’s multiple comparison tests. Comparisons between stimulation sites, evoked dopamine responses, and sucrose consumption between groups were compared using One-Way ANOVA with post hoc Tukey’s or Holm-Sidak multiple comparison’s tests. Press rates in VI60, VI30, and devaluation probes, as well as time on stimulation side in place preference, baseline velocity, changes in velocity, and autocorrelations were compared using unpaired student's t-tests. Pearson’s Correlation was utilized for all correlations. Statistical outliers were determined using the ROUT (robust regression followed by outlier identification) method (Q = 0.5%) in GraphPad Prism 7.04 (GraphPad), and were removed prior to statistical analyses. Finally, mice lacking ostensible viral expression in the striatum were excluded prior to analysis. For all tests, significance was defined as *p* ≤ 0.05.

## Supplementary Information

Supplementary Information.
