# Supplementary material for: Optogenetic stimulation of striatal patches modifies habit formation and inhibits dopamine release
Source: Sci Rep. 2021 Oct 6;11:19847. doi: 10.1038/s41598-021-99350-5 (PMC8494762; doi:10.1038/s41598-021-99350-5)
Supplement: Supplementary file 1 — Supplementary Information. [file 41598_2021_99350_MOESM1_ESM.docx]

**Optogenetic stimulation of striatal patches modifies habit formation and inhibits dopamine release**

Nadel, J.A.^1, #^, Pawelko S.S.^1, #^, Scott J.R.^1^, McLaughlin, R.^1^, Fox M.^1^, Ghanem, M.^1^, van der Merwe, R.^1^, Hollon, N.G.^2^, Ramsson, E. S.^3^, Howard, C.D.^1,^*

^#^These authors contributed equally to this work

*Corresponding Author

Email: [choward@oberlin.edu](mailto:choward@oberlin.edu)

^1^ Neuroscience Department, Oberlin College, Oberlin, OH, USA

^2^ Molecular Neurobiology Laboratory, The Salk Institute for Biological Studies, La Jolla, CA, USA.

^3^ Department of Biomedical Science, Grand Valley State University, Allendale, MI, USA.

**Supplemental Figure 1**


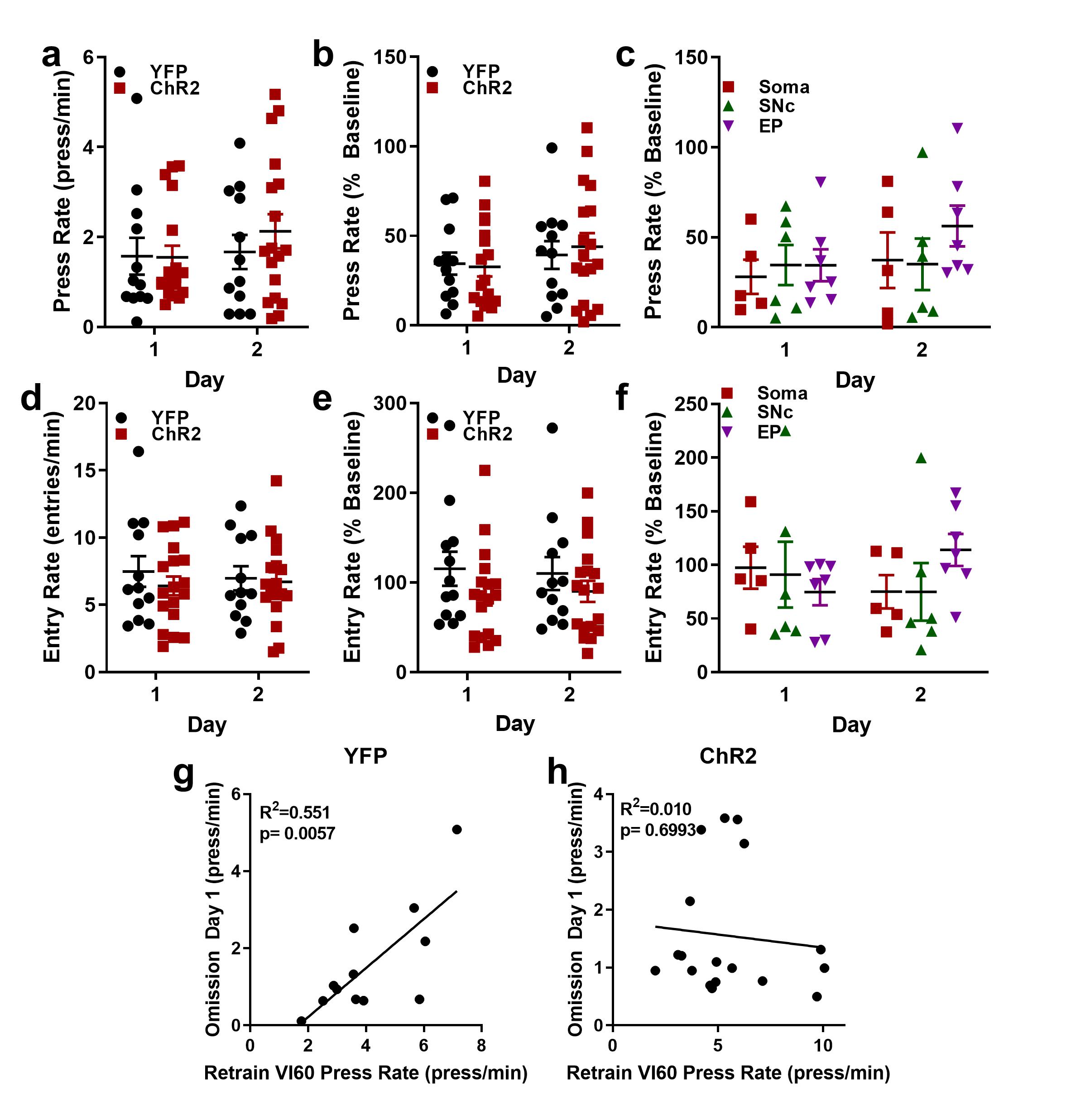


**Supplemental Figure 1. Effects of optogenetic patch stimulation during learning on omission.** (**a**) Average press rates during omission probes for YFP control and ChR2 mice across days (two-way repeated measures ANOVA, no significant effects of time, F_(1, 28)_ = 3.169, p = 0.0859; group, F_(1, 28)_ = 0.2111, p = 0.6495; or interaction, F_(1, 28)_ = 1.624 p = 0.2130). (**b**) Average press rates normalized to responding during VI60 retraining during omission probes (two-way repeated measures ANOVA, trending effect of time, F_(1, 28)_ = 3.647, p = 0.0665; no significant effect of group, F_(1, 28)_ = 0.02412, p = 0.8777; or interaction F_(1, 28)_ = 0.5788, p = 0.4531). (**c**) Same data as **b**, but broken into fiber optic placement groups (two-way repeated measures ANOVA, significant effect of time, F_(1,26)_ = 4.559, p = 0.0423, no significant effect of group, F_(2, 15)_ = 0.3990, p = 0.6779; or interaction F_(2, 15)_ = 1.708, p = 0.2147). (**d**) Average head entry rates during omission across days (two-way repeated measures ANOVA, no significant effects of time, F_(1, 28)_ = 0.02714, p = 0.8703; group, F_(1, 28)_ = 0.4231, p = 0.5207; or interaction, F_(1, 28)_ = 0.4071, p = 0.5286). (**e**) Average entry rates normalized to baseline entry rates in VI60 retraining (two-way repeated measures ANOVA, no significant effects of time, F_(1, 28)_ = 0.01001, p = 0.9210; group, F_(1, 28)_ = 1.558, p = 0.2223; or interaction, F_(1, 28)_ = 0.3356, p = 0.5670). (**f**) Same as **e**, but broken into fiber optic placement groups (two-way repeated measures ANOVA, significant time x group interaction, F_(2, 15)_ = 5.780, p = 0.0138, no significant *post hoc* Tukey tests, all p ≥ 0.674). (**g-h**) Correlation of omission press rate on day 1 vs. VI60 retraining day immediately preceding omission for YFP (**g**) or ChR2 (**h**) mice. Data are mean ± SEM.

**Supplemental Figure 2**


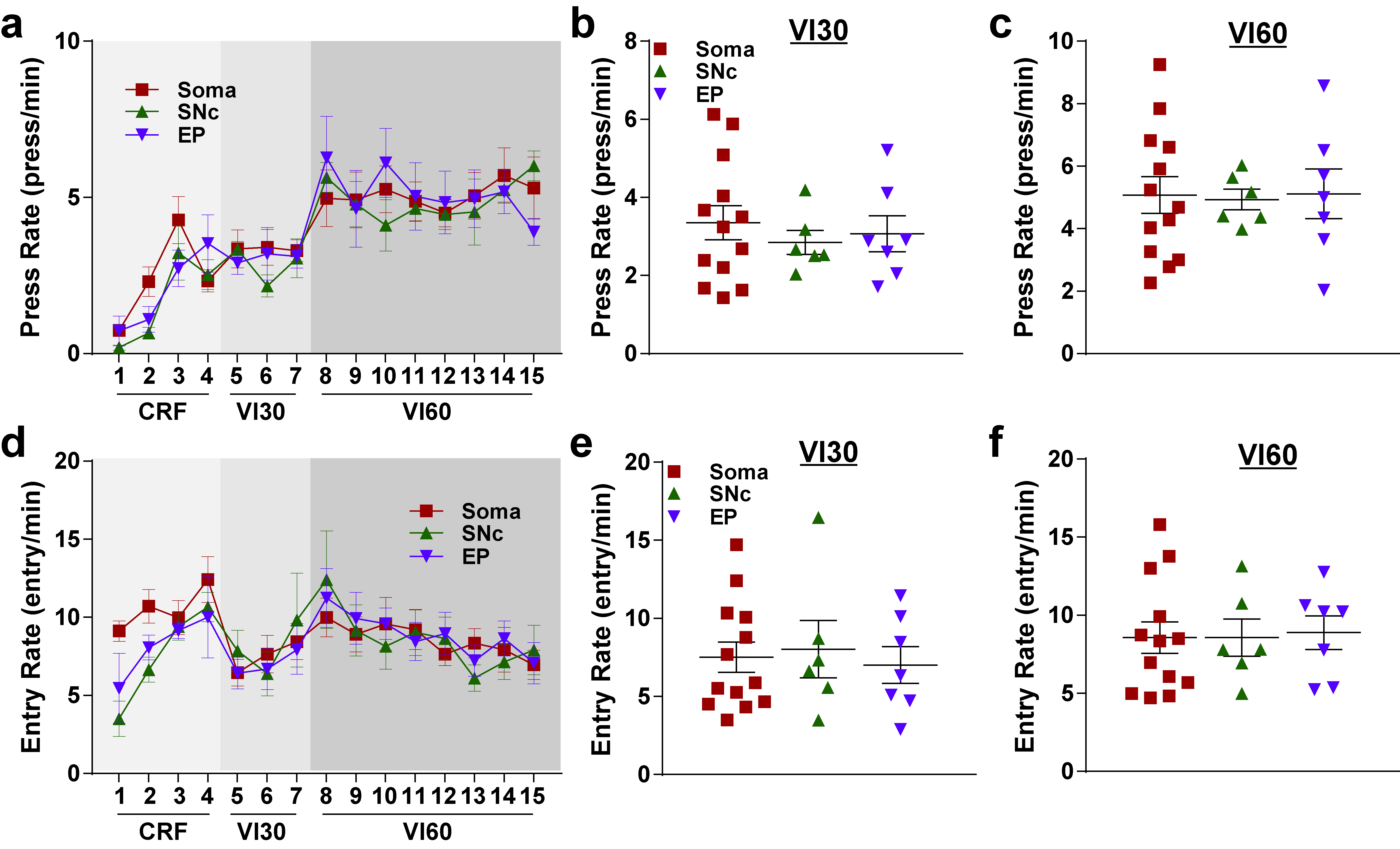


**Supplemental Figure** **2. Optogenetic patch stimulation during variable interval training by implantation site group.** (**a**) Average press rates across continuous reinforcement (CRF), variable interval 30 (VI30), and variable interval 60 (VI60) training by day (two-way repeated measures ANOVA, significant effect of time, F_(14,322)_ = 16.28, p < 0.0001, no significant effects of group, F_(2, 23)_ = 0.1693, p = 0.8453; or interaction, F_(28, 322)_ = 0.7915, p = 0.7679). (**b-c**). Average press rates across all VI30 (**b**; one-way ANOVA, F_(2,23)_ = 0.3060, p = 0.7393) and VI60 (**c**; one-way ANOVA, F_(2,23)_ = 0.01698, p = 0.9832) days. (**d**). Average head entry rates across training by day (two-way repeated measures ANOVA, significant effect of time, F_(14,322)_ = 6.141, p < 0.0001, no significant effects of group, F_(2, 23)_ = 0.1504, p = 0.8612; and a trending interaction, F_(28, 322)_ = 1.433, p = 0.0760). (**e-f**) Average entry rate across all VI30 (**e**; one-way ANOVA, F_(2,23)_ = 0.1248, p = 0.8833) and VI60 (**f**; one-way ANOVA, F_(2,23)_ = 0.02407, p = 0.9762) days.

**Supplemental Figure 3**


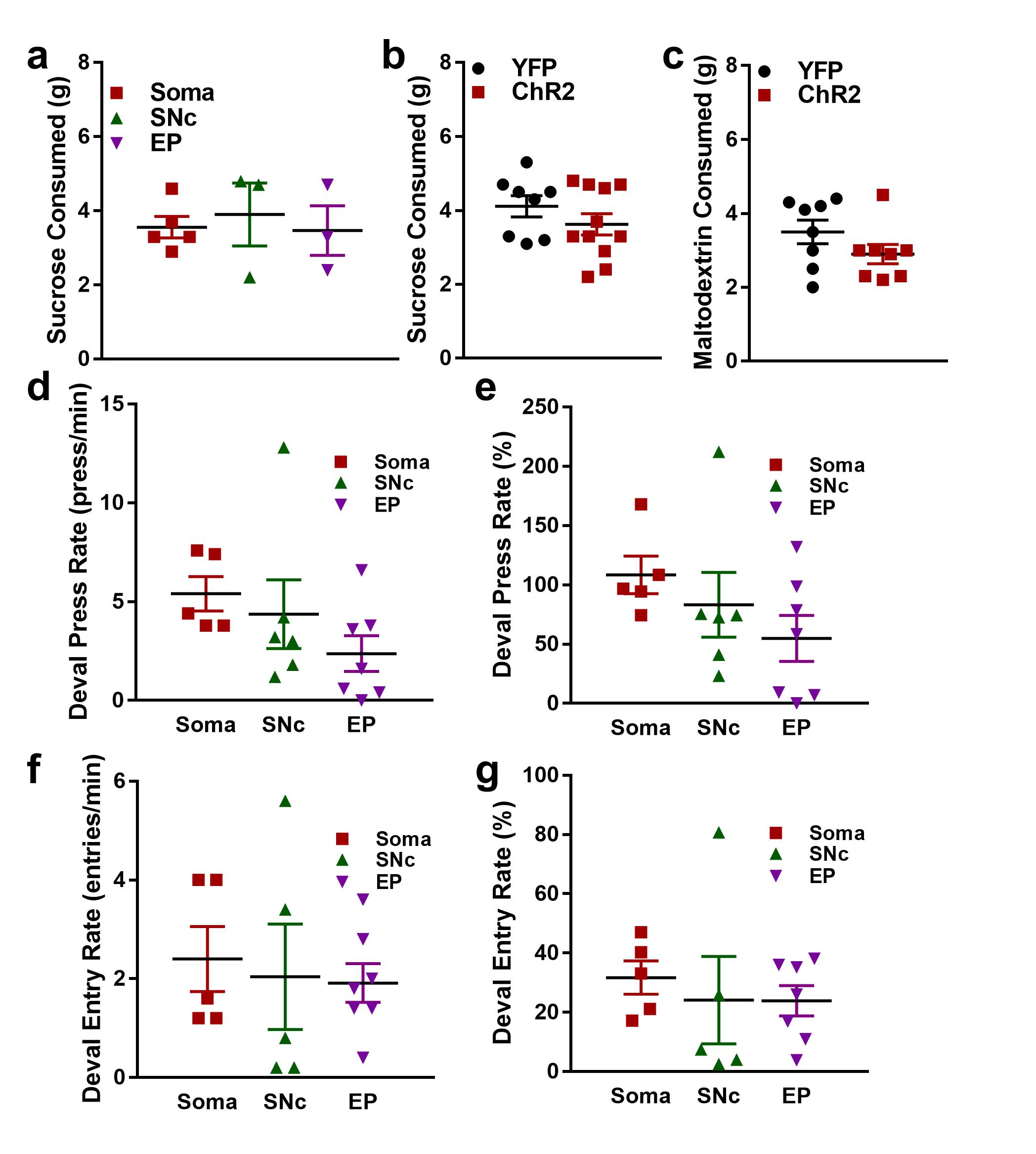


**Supplemental Figure** **3. Sucrose consumed during free access and press and entry rates in devaluation by implantation site group.** (**a-b**) Average sucrose consumed during 1h free access by implantation site group (**a**; one-way ANOVA, F_(2,8)_ = 0.1486, p = 0.8642) and collapsed into YFP/ChR2 groups (**b**; unpaired t-test, t_17_ = 1.166, p = 0.2596). (**c**) average maltodextrin consumed during 1h free access (unpaired t-test, t_14_ = 1.449, p = 0.1693). (**d-e**) Average press rate (**d**; one-way ANOVA, F_(2,15)_ = 1.541, p = 0.2461) and average press rates normalized to responding across VI60 training (**e**; one-way ANOVA, F_(2,15)_ = 1.484, p = 0.2582). (**f-g**) Average head entry rates (**f**; one-way ANOVA, F_(2,14)_ = 0.130, p = 0.8785) and entry rates normalized to baseline VI60 responding (**g**; one-way ANOVA, F_(2,14)_ = 0.244, p = 0.7865). Data are mean ± SEM.
 **Supplemental Figure 4**

**
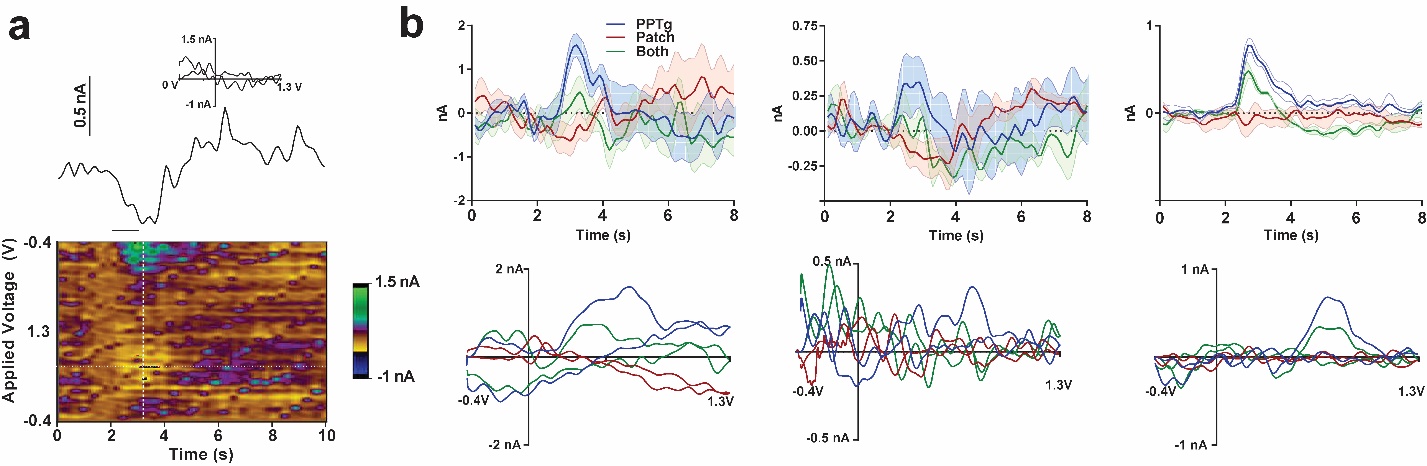

Supplemental Figure 4. Average data from each replicate in FSCV experiments.** (**a**) Representative decrease in current recorded during an “opto” trial (stimulation of patch terminals only). The line shows recorded current relative to stimulation delivery (straight line below current trace) above a pseudo-color plot. The color plot shows current collected (in color) at each waveform scan (y-axis) and across time (x-axis). INSET: a “cyclic voltammogram” collected at the vertical white dotted line on the pseudo-color plot. (**b**) Average current (top) and cyclic voltammograms (bottom) recorded across three trials for each of the three stimulation conditions. Each current and cyclic voltammogram plot is from an individual FSCV experiment.
